# Alkaloids in *Erythrina* by UPLC-ESI-MS and *In Vivo* Hypotensive Potential of Extractive Preparations

**DOI:** 10.1155/2015/959081

**Published:** 2015-08-18

**Authors:** Liara Merlugo, Marí C. Santos, Liane S. Sant'Anna, Everson W. F. Cordeiro, Luiz A. C. Batista, Silvia T. S. Miotto, Cássia V. Garcia, Cleci M. Moreira, Andreas S. L. Mendez

**Affiliations:** ^1^Programa de Pós-graduação em Bioquímica, Universidade Federal do Pampa, BR 472 Km 585, Caixa Postal 118, 97508-000 Uruguaiana, RS, Brazil; ^2^Laboratório de Desenvolvimento e Controle de Qualidade de Medicamentos, Universidade Federal do Pampa, BR 472 Km 585, Caixa Postal 118, 97508-000 Uruguaiana, RS, Brazil; ^3^Laboratório de Fisiologia Cardiovascular, Universidade Federal do Pampa, BR 472 Km 585, Caixa Postal 118, 97508-000 Uruguaiana, RS, Brazil; ^4^Departamento de Botânica, Universidade Federal do Rio Grande do Sul, Porto Alegre, RS, Brazil; ^5^Faculdade de Farmácia, Universidade Federal do Rio Grande do Sul, Avenida Ipiranga 2752, 90610-000 Porto Alegre, RS, Brazil

## Abstract

*Erythrina* species are used in popular medicine as sedative, anxiolytic, anti-inflammatory, and antihypertensive. In this work, we investigated the chemical composition of extracts obtained from leaves of *E. falcata* and *E. crista-galli*. The hypotensive potential of *E. falcata* and the mechanism of action were also studied. The extracts were obtained by maceration and infusion. The total content of phenolic compounds and flavonoids was estimated by spectrophotometric methods. The chemical constituents were studied performing a chromatographic analysis by UPLC-ESI-MS. For *in vivo* protocols, blood pressure and heart rate were measured by the invasive hemodynamic monitoring method. Different concentrations of extracts and drugs such as L-NAME, losartan, hexamethonium, and propranolol were administrated i.v. The results of total phenolic contents for *E. falcata* and *E. crista-galli* were 1.3193–1.4989 mgGAE/mL for maceration and 0.8771–0.9506 mgGAE/mL for infusion. In total flavonoids, the content was 7.7829–8.1976 mg RE/g for maceration and 9.3471–10.4765 RE mg/g for infusion. The chemical composition was based on alkaloids, suggesting the presence of erythristemine, 11*β*-methoxyglucoerysodine, erysothiopine, 11*β*-hydroxyerysodine-glucose, and 11-hydroxyerysotinone-rhamnoside. A potent dose-dependent hypotensive effect was observed for *E. falcata*, which may be related to the route of *β*-adrenergic receptors.

## 1. Introduction

Hypertension is a global health problem whose increased incidence can lead to the development of many chronic diseases, as well as collaborating in the progression of chronic kidney disease, and contribute to cardiovascular morbidity and mortality [1]. Although there is a wide range of antihypertensive drugs used in the control of hypertension, a successful treatment becomes even harder to achieve [2], so the knowledge of medicinal plants, their composition, and mechanism of action could have a vital role in the discovery of new products as therapeutic agents.

The genus* Erythrina* (Fabaceae) consists of 110 species, distributed in the tropical regions of America, Africa, Asia, and Oceania [3, 4]. Phytochemical studies of its different species showed a chemical constitution by alkaloids, flavonoids, pterocarpans, and cumarines [5–9]. Species as* E. velutina, E. mulungu, E. crista-galli*, and* E. falcata* demonstrated the predominance of alkaloids and flavonoids in their composition [10–14]. The alkaloids present in* Erythrina* species have attracted much attention from researchers, since they represent the main source of the tetracyclic alkaloids of* Erythrina* type and have similar activities of the curare, causing muscle paralysis [15].

In popular medicine, these species are used for the treatment of respiratory diseases, as well as anxiolytic, sedative, anti-inflammatory, and antihypertensive [11, 16]. Scientific studies showed different activities for these species such as antibacterial, antifungal, and anti-inflammatory [17–19]. Lehman [20] tested alcoholic extracts of* E. americana* and proved its hypotensive effect in rats, rabbits, and dogs. Alkaloids obtained from this species also demonstrated hypotensive activity [21].

The present study aimed to investigate the chemical composition of the species* E. falcata* and* E. crista-galli* by UPLC-ESI-MS and to evaluate the hemodynamic parameters in rats, purposing to elucidate the involved mechanism of action for* E. falcata* extracts.

## 2. Materials and Methods

### 2.1. Plant Material

*Erythrina crista-galli* was collected in May 2013 in Uruguaiana, RS (Brazil).* Erythrina falcata* was collected in Porto Alegre, RS (Brazil), in February 2014. The materials were identified and a voucher specimen of each (*Erythrina crista-galli*, ICN 183940;* Erythrina falcata* Benth., ICN 177665) was deposited at the ICN Herbarium (Instituto de Biociências, UFRGS, Avenida Bento Gonçalves, 9500, Prédio 43433, CEP 91501-970, Porto Alegre, RS, Brazil).

### 2.2. Extracts Preparation

Leaves were selected and dried at a temperature of 40°C for five days. After that, they were grounded and submitted to exhaustive extraction processes by maceration using 40% ethanol as solvent (1 : 10, plant : solvent) and by infusion using distilled water at a temperature of 100°C (1 : 15, plant : solvent).

For chromatographic assays, extracts obtained by maceration process of both species were used. To reduce the impurities, extracts passed through a liquid-liquid extraction method by affinity solvents with increasing polarity (hexane, dichloromethane, ethyl acetate, and methanol) and for further analysis they were filtered on a membrane of 0.45 micrometers.

For biological analyses macerations were used and infusions were obtained from* E. falcata*, which were oven-dried and reduced to powder for subsequent redilution in 0.9% saline solution for administration.

### 2.3. Total Phenolic Content

The total phenolic content was determined by Folin-Ciocalteu colorimetric method with some modifications [22]. For the analyses, aliquots of 100 *μ*L of the samples and standard solutions were transferred to test tubes, where 500 *μ*L of Folin-Ciocalteu reagent and 6 mL of distilled water were added. Then, they were agitated and left at rest for 1 minute. After alkalizing the medium, 2 mL of Na_2_CO_3_ solution 15% was added, and the volume was completed to 10 mL with distilled water. After 30 minutes at room temperature and being protected from light, samples were analysed in a spectrophotometer Perkin Elmer UV-VIS Lambda 35 (Norwalk, CT, USA) at 750 nm. The total phenolic content was expressed in milligrams of gallic acid equivalent per mL of sample (mgGAE/mL).

### 2.4. Total Flavonoid Content

The total flavonoid content was determined by the method described by Chang et al. [23] with some modifications. In a 25 mL flask 500 *μ*L of sample and 500 *μ*L of AlCl_3_ 0.5% were added and the volume was completed with water. It was incubated for 40 minutes at room temperature and protected from light and reading was done in UV-VIS spectrophotometer Lambda 35 Perkin Elmer (Norwalk, CT, USA) at a wavelength of 415 nm. The results were expressed in mg/g equivalents of rutin.

### 2.5. UPLC-MS

For the protocols a reverse-phase system was performed, using the following conditions: fast C18 analytical column Shim-pack XR-ODS (50 × 2 mm, 2.1 *μ*m); mobile phase consisted of acetonitrile : methanol 4 : 1 (v/v) and water with 0.1% formic acid (pH 3.0) with a flow rate of 0.2 mL/min; DAD detection at 340 nm; injection volume of 5.0 *μ*L. The elution gradient was as follows: 0% solvent A (0–2.5 min), 0–5% A (2.5–7.5 min), 5–10% A (7.5–10 min), 10–15% A (10–12.5 min), 15–20% A (12.5–15 min), 20–25% A (15–17.5 min), 25% A (17.5–20 min), 25–60% A (20–27.5 min), 60% A (27.5–30 min), and 60–0% A (30–35 min).

Chromatographic analyses were performed using an Acquity UPLC system (Waters Co., MA, USA) equipped with binary solvent delivery system and autosampler. The DAD-UV and mass spectrometer EM Q-TOF Micro-Micromass (Waters Co., MA, USA) detectors were employed, with MassLynx v. 4.1 data acquisition software.

The mass spectrometry analyses were performed in positive-ion mode and operated according to the following conditions: collision energy 4.0 eV; temperature of electrospray source and desolvation gas 100°C and 120°C, respectively; capillary voltage 3000 V; sample cone 40 V; extraction cone 3.0 V; N_2_ was used as the nebulizing. Mass spectra were recorded by using full scan mode in the range of* m/z* 200–800.

### 2.6. Hypotensive Activity and Mechanism of Action from* E. falcata* Extracts

#### 2.6.1. Animals

Three-month-old male Wistar rats, weighing around 300 g and purchased from the animal house of the Universidade Federal de Santa Maria, were used. They were kept in cages with water available* ad libitum*, maintaining the temperature controlled at 20–22°C with dark/light cycle of 12 hours.

The experimental protocols followed the International Principles for Research Involving Animals (Geneva) (Brazilian legislation by law number 11.794/2008 (procedures for the scientific use of animals), Decree 24.645/34 (animal rights), approved by the Ethics Committee on Animal Use (CEUA), Universidade Federal do Pampa (Protocol 030/2013)).

#### 2.6.2. Hemodynamic Parameters

Rats were anesthetized with urethane (1.4 mg/kg, i.p.) and submitted to surgery for catheterization of the carotid artery (to measure the hemodynamic parameters) and the jugular vein (for administration of the extracts and drugs). Anesthesia was assessed by responsiveness to painful stimuli and when necessary the anesthetic dose was supplemented. For the catheterization, polyethylene catheters (PE 10, Clay-Adams) filled with heparinized saline (50 IU/mL) were used. The arterial catheter was connected to a pressure transducer coupled to an analogue to digital converter (Biopac Systems MP150, Inc.; CA).

#### 2.6.3. Extract Curve

After 30 minutes of stabilization on the equipment, in order to evaluate the effects of the extracts on hemodynamic parameters, a rising curve was performed. 0.5, 2.5, 5, 10, 25, 50, 75, and 100 mg/kg of extract with a volume of 0.2 mL of saline were administered every 10 minutes (*n* = 8). Records of systolic blood pressure (SBP), diastolic blood pressure (DBP), and heart rate (HR) were made.

#### 2.6.4. Evaluation of Mechanism of Action from Extracts

In order to investigate some possible mechanisms involved in the hypotensive response of the extracts, some drugs were tested after 30 minutes of stabilization: L-NAME, an NO synthesis inhibitor (30 mg/kg, *n* = 8); losartan, AT1 receptor antagonist of angiotensin II (10 mg/kg, *n* = 8); hexamethonium, ganglionic blocker (20 mg/kg, *n* = 8); and propranolol, *β*-adrenergic blocker (5 mg/kg, *n* = 8) [24–27]. After drug administration, a new stabilization period was done and then the extract was administrated (25 mg/kg and 50 mg/kg). Hemodynamic records of SBP, DBP, and HR were then made.

#### 2.6.5. Statistical Analysis

Data were expressed as mean ± standard error of the mean (SEM) and values were analyzed by analysis of variance (ANOVA) followed by* post hoc* Tukey. Significance was set at *p* < 0.05.

## 3. Results and Discussion

### 3.1. Characterization of Plant and Extracts

The water content determination of* E. falcata* and* E. crista-galli* presented values of 64.54% and 63.77%, respectively. The values of total phenolic and total flavonoid content are expressed in Table 1, whose results ranged 0.8771–1.4989 mg GAE/mL and 7.7829–10.4765 mg RE/g, respectively.

In a study involving the species* E. indica*, the phenolic and flavonoids content were quantified for aqueous and methanolic extracts obtained by maceration [28]. The values were 24.91 and 25.62 mg GAE/g for total phenolic 357.55 and 524.22 mgRE/g for total flavonoids, respectively. Sowndhararajan et al. [29] also evaluated the phenolic content in* E. indica* for methanolic extracts obtained by maceration of stem, bark, and leaves, demonstrating 412.8 and 184.3 mg GAE/g extract. Another species,* E. velutina*, was evaluated in terms of total phenolic content in ethanolic and hydroethanolic extracts of seeds, where the levels found were 423.6 and 236.3 mg catechin equivalents/100 g, respectively [30].

Our results showed the presence of these chemical classes in the studied medicinal plants, although differences could be observed when compared to published data, probably related to environmental factors, collection locations, experimental procedures (extractive processes), and quantitative values expression.

### 3.2. UPLC-MS

**Figure 1:**
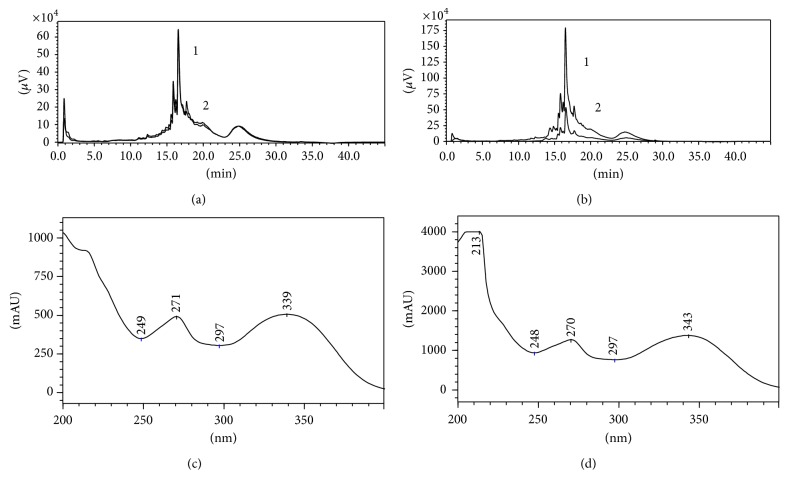
Illustrative chromatograms obtained from analysis of* Erythrina* leaves extracts, with detection at 280 nm. (a)* E. falcata*: 1: crude hydroethanolic extract at 40%; 2: ethanolic residual fraction. (b)* E. crista-galli*: 1: crude hydroethanolic extract at 40%; 2: ethanolic residual fraction. (c) UV profile of major chromatographic peak from* E. falcata*. (d) UV profile of major chromatographic peak from* E. crista-galli*.

For a better separation profile in the chromatographic run, the analysis was performed by continuous monitoring at 280 and 340 nm, considering as reference for alkaloids and phenolic compounds. In order to improve major peaks, these extracts passed through a purification process by liquid-liquid separation involving increasingly polar solvents (hexane, dichloromethane, ethyl acetate, and methanol). We can observe in Figure 1 that the residual ethanol fraction presented a similar profile of separation in the range of 15 to 20 min for both species, when compared to crude extract, although the chemical constituents were detected in major concentrations after purification. Based on these results, the residual ethanol fraction was submitted to analysis by UPLC-MS.

**Figure 2:**
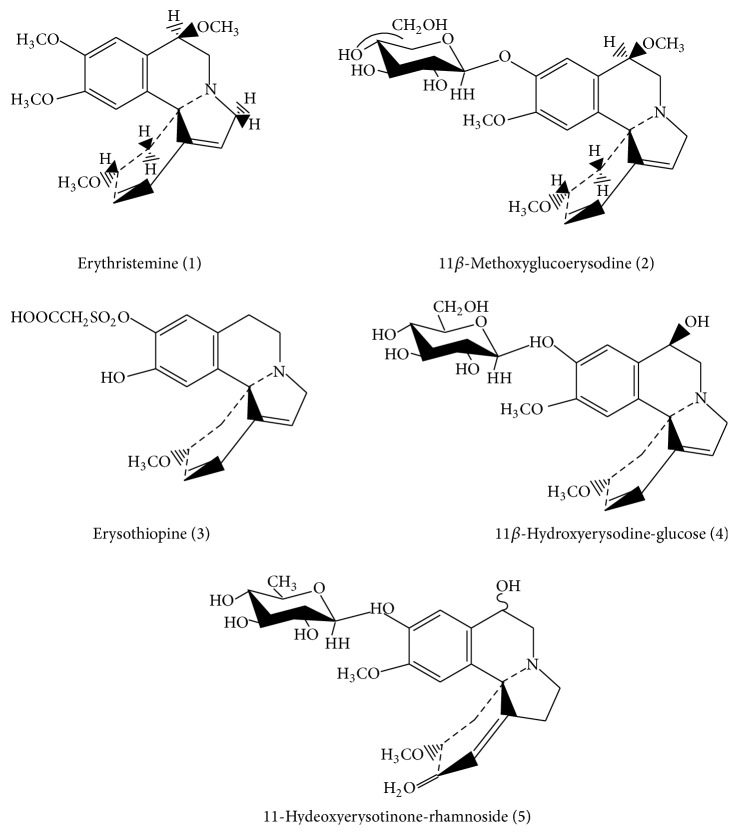
Chemical structures of alkaloids suggested for extracts obtained from* E. falcata* and* E. crista-galli*.

The species of the genus* Erythrina* are known for their significant bioproduction of alkaloids, which is the main source of tetracyclic* Erythrina* alkaloids [31]. Based on reported data, a detailed analysis of mass spectra and their respective fragments was performed (see Supplementary Material available online at http://dx.doi.org/10.1155/2015/959081). The careful interpretation was conducted and allowed to suggest the presence of alkaloids derivatives, whose description is illustrated in Table 2. The respective chemical structures are presented in Figure 2.

The presence of these alkaloids has been cited in some works with several species of* Erythrina* [31–36]. In our study, the presence of the alkaloid erythristemine in both species can be suggested by analyzing the molecular ion [M+H]^+^ at* m/z* 344 and its fragments. This alkaloid has been found in the flowers of* E. x bidwillii*, a species located in the desert regions [37]. This compound was also reported in* E. lysistemon* [31]. Juma and Majinda [32] isolated this alkaloid from flowers, pods, and seeds of* E. lysistemon*, and also assessed its antioxidant activity.

For the molecular ion [M+H]^+^ at* m/z* 492 and respective fragments, it is possible to verify a loss of 162 Da, which requires our attention because it is possibly a fragment of hexose [38]. Thus, the presence of the alkaloid 11*β*-methoxyglucoerysodine is suggested. This derivative has been described in* E. lysistemon* according to study by Amer et al. [33] that evaluated the composition of the fruits of this species.

Equivalent fragments of sugars were also observed in other spectra, suggesting possible alkaloids that may be attached to these fragments as 11*β*-hydroxyerysodine linked to a hexose which corresponds to 162 Da or even 11-hydroxyerysotinone linked to a rhamnoside fragment, equivalent to 147 Da. These alkaloids, as aglycones, have been described in species of* Erythrina*. 11*β*-hydroxyerysodine has been described in* E. lysistemon* [31] and 11-hydroxyerysotinone in* E. macrophylla* and* E. americana* [34, 35].

Considering the molecular ion [M+H]^+^ at* m/z* 408, the presence of erysothiopine alkaloid is suggested, which was also found in* E. glauca* seeds [36]. Additionally, this compound is described as one of natural sources of agonists and antagonists of nicotinic acetylcholine receptors [39].

### 3.3. Hemodynamic Parameters

#### 3.3.1. Evaluation of* E. falcata* Extract Hemodynamic Curve

According to folk medicine genus* Erythrina* is effective for treatment of hypertension. Ethnobotanical studies also have mentioned the therapeutic use as hypotensive [40–44]. In our study, we consider some literature data that reports the probable hypotensive activity in medicinal species* E. falcata* [40]. Moreover, this species is not frequently explored scientifically, despite its popular use in South of Brazil which indicates a promisor* in vivo* potential. This context justifies our choice for applying* in vivo* protocols only for* E. falcata*.

**Figure 3:**
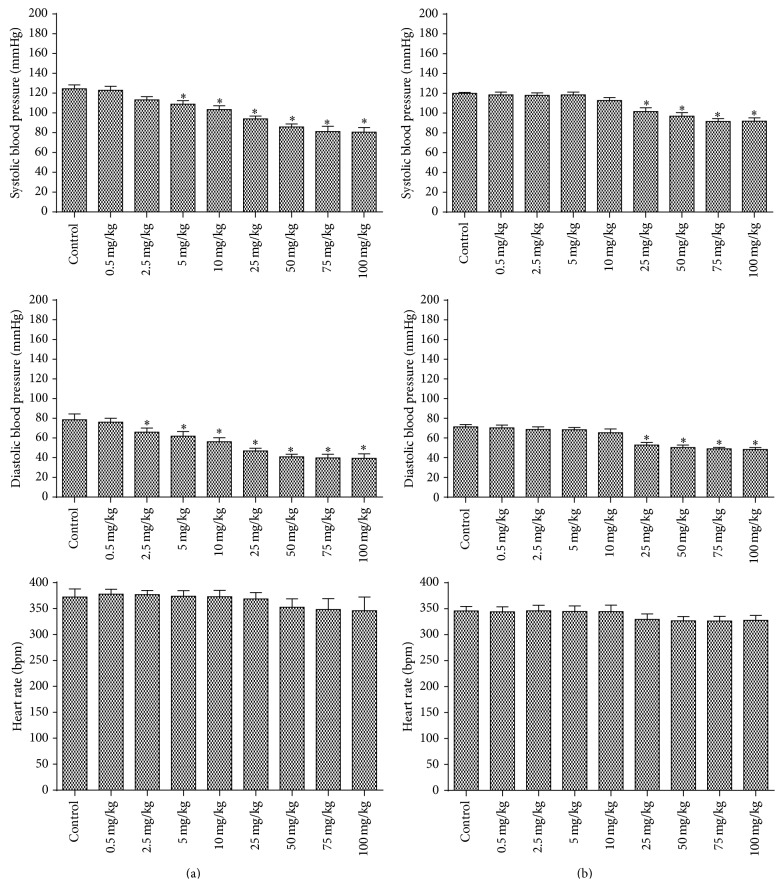
Different doses effect of* E. falcata* (a) infusion and (b) maceration on hemodynamic parameters in rats. ^*∗*^ *p* < 0.05 versus control (*n* = 8).

In the administration curve of infusion and maceration extracts from* E. falcata*, an effective and dose-dependent hypotensive action can be observed without affecting the HR (Figure 3). The blood pressure decreased by infusion from doses of 5 mg/kg and 2.5 mg/kg for SBP and DBP, respectively. The maceration extract presented action since the dose of 25 mg/kg for SBP and DBP.

Another species of* Erythrina* already showed hypotensive effects. A study by Lehman [20] has shown that alcoholic extracts obtained from* E. americana*, when administered intravenously to dogs, rabbits, and rats, caused a decrease in blood pressure from 10 to 20%. Hargreaves et al. [21] also verified the effects of* E. americana*, demonstrating that it has similar alkaloids suggested in this work and, between various effects, it showed hypotensive activity.

As described in the chemical characterization of the extracts, the content of alkaloids could be contributing on the hypotensive effect. Although alkaloids have a wide range of pharmacological properties, some studies indicated their cardiovascular benefits [45–47]. The erythrinic alkaloids already have many effects described such as antioxidant, anticarcinogenic, anxiolytic, analgesic, and anticonvulsant [48–51]; however, the alkaloids suggested for* E. falcata* were not well studied pharmacologically; we could expect that these bioactive substances obtained from these genera have important pharmacological potentials.

Despite some reports, the present work is the first that describes the hypotensive activity of hydroethanolic and aqueous extracts of* E. falcata,* which is extremely important since the incidence of hypertension and use of medicinal plants has been increasing worldwide, generating, thus, future potential phytotherapy and also ensuring the safety of its use by the population.

#### 3.3.2. Evaluation of Mechanism of Action from* E. falcata* Extracts

**Figure 4:**
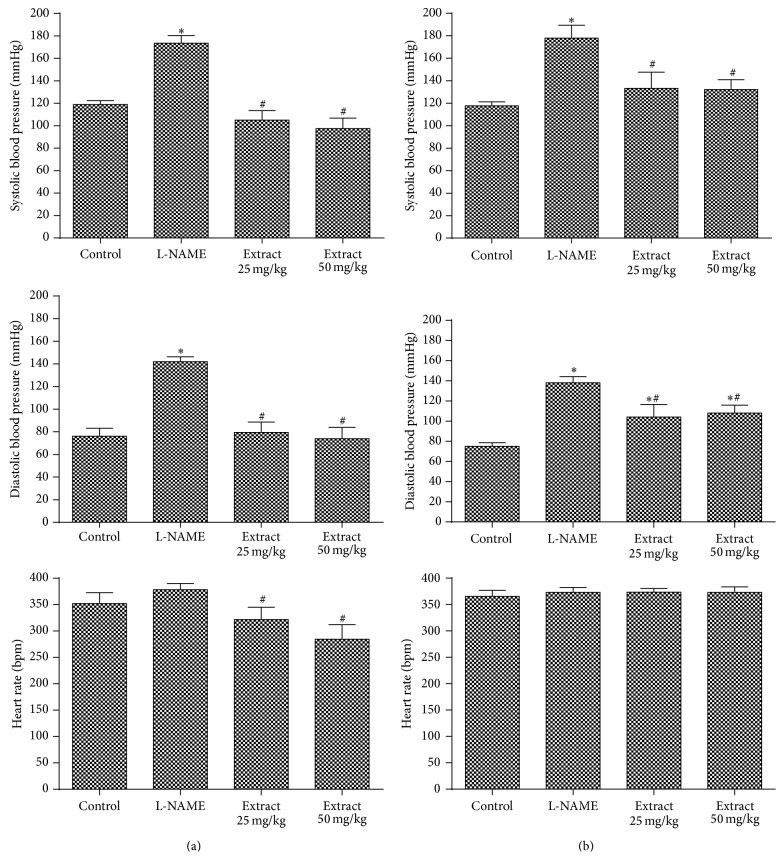
Evaluation of hemodynamic parameters in rats treated with doses of 25 mg/kg and 50 mg/kg of* E. falcata* (a) infusion and (b) maceration in the presence of L-NAME (30 mg/kg). ^*∗*^ *p* < 0.05 versus control and ^#^ *p* < 0.05 versus L-NAME (*n* = 8).

To investigate the possible mechanisms involved in the hypotensive activity of the extracts, different specific drugs, such as L-NAME, losartan, hexamethonium, and propranolol, were administered. L-NAME is an analogue of arginine and competes for NO-synthase generating a prolonged increase in blood pressure and resulting in gross inhibition of NO synthesis [52]. It is widely used in experiments with rats as a model of systemic hypertension [53–57]. After L-NAME, when the infusion extract is administered, SBP, DBP, and HR decreased significantly at both doses (Figure 4(a)). When the maceration extract was administered, SBP and DBP decreased in both doses without changing HR (Figure 4(b)). These data demonstrate that hypotensive action do not have relation to NO pathway, since there was a decrease in blood pressure after administration of L-NAME. Similar results were observed in studies of Kang et al. [54] and Kang et al. [55] that evaluated the effects of* Cudrania tricuspidata* and* Fritillaria ussuriensis* extracts on blood pressure of L-NAME-induced hypertension rats.

**Figure 5:**
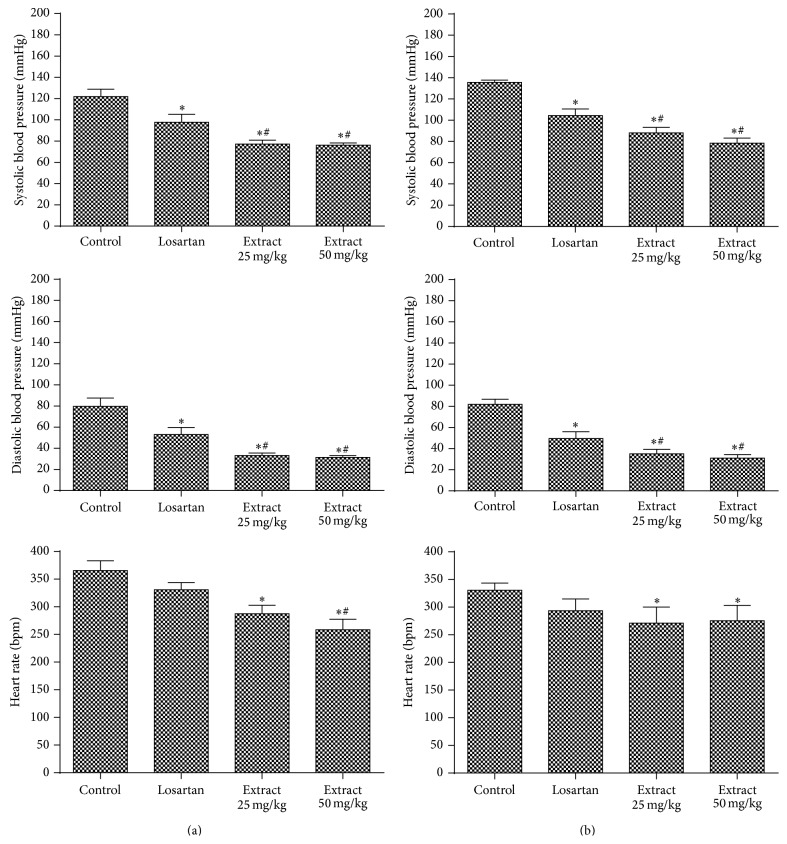
Evaluation of hemodynamic parameters in rats treated with doses of 25 mg/kg and 50 mg/kg of* E. falcata* (a) infusion and (b) maceration in the presence of losartan (10 mg/kg). ^*∗*^ *p* < 0.05 versus control and ^#^ *p* < 0.05 versus losartan (*n* = 8).

Losartan causes hypotension through blocking the renin-angiotensin system by selectively antagonizing the receptors for angiotensin II, subtype AT1 [58]. The administration of the extracts, after AT1 receptor blockade, caused a significant decrease in SBP, DBP, and HR at both doses (Figure 5), indicating that this mechanism would not be involved in the hypotensive response. The results of Jiménez-Ferrer et al. [59] differ from our study, but they evaluated the antihypertensive activity of* Salvia elegans* extracts compared with losartan as positive control.

**Figure 6:**
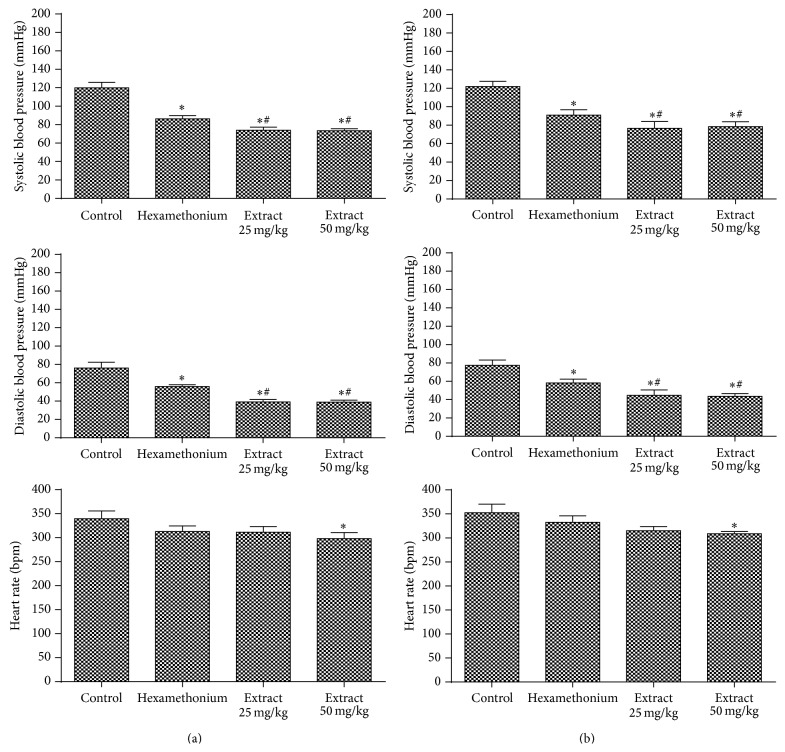
Evaluation of hemodynamic parameters in rats treated with doses of 25 mg/kg and 50 mg/kg of* E. falcata* (a) infusion and (b) maceration in the presence of hexamethonium (20 mg/kg). ^*∗*^ *p* < 0.05 versus control and ^#^ *p* < 0.05 versus hexamethonium (*n* = 8).

The hexamethonium acts on the sympathetic ganglionic blockade, causing decreasing in the blood pressure from arteriolar vasodilatation and cardiovascular reflex blockade [52, 60]. The administration of the extracts after this drug caused a significant reduction in SBP and DBP at both doses. In HR a significant reduction was observed only at 50 mg/kg dose (Figure 6). So this pathway is also not related to the mechanism of action. Study by Suárez et al. [61] evaluated* Pimenta dioica* extract in SHR rats using hexamethonium. The extract's hypotensive response decreased significantly with ganglionic blocking, different from our results.

**Figure 7:**
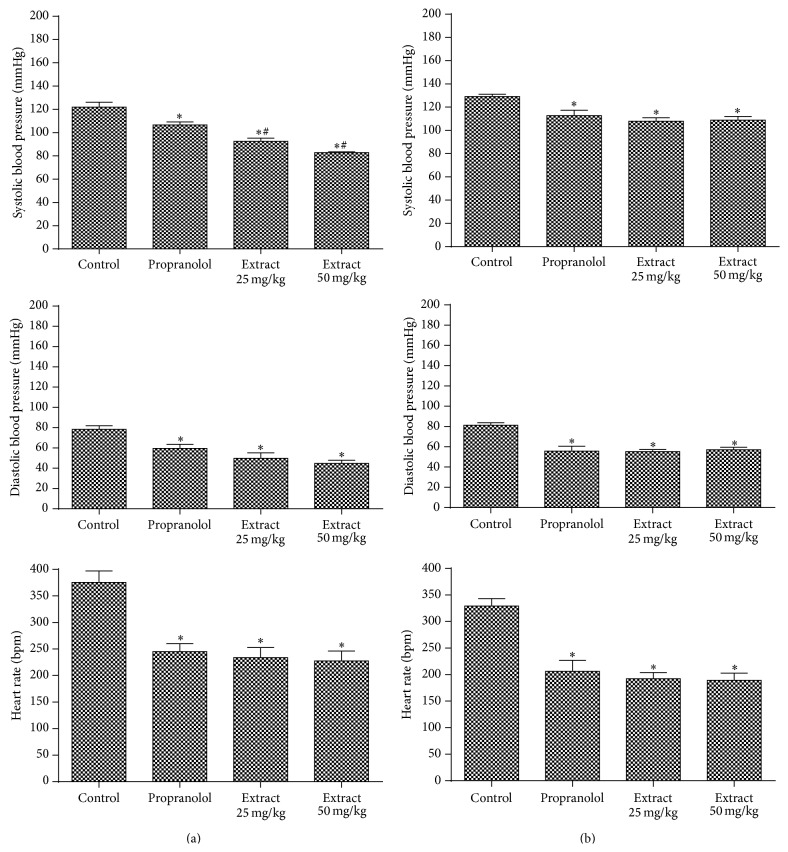
Evaluation of hemodynamic parameters in rats treated with doses of 25 mg/kg and 50 mg/kg of* E. falcata* (a) infusion and (b) maceration in the presence of propranolol (5 mg/kg). ^*∗*^ *p* < 0.05 versus control and ^#^ *p* < 0.05 versus propranolol (*n* = 8).

Another drug used was propranolol, which is a potent antagonist nonselective of *β*-adrenergic receptors, acting on *β*1 and *β*2 receptors, causing, among other effects, decrease in blood pressure [52]. After the drug, the infusion administration reduced SBP at both doses, but DBP and HR were not decreased in comparison to losartan (Figure 7(a)). Already the administration of maceration extract, SBP, DBP, and HR reduced only in comparison with the control (Figure 7(b)). So we suggest that the hypotensive mechanisms from extracts may be related to the route of *β*-adrenergic receptors.

Nembo et al. [62] investigated the chronotropic effect of aqueous extract of* E. senegalensis* and verified that *β*-adrenergic pathway could be involved in the HR decrease caused by the extract in rats.

Other studies with plant extracts also pointed *β*-adrenergic pathway as mechanism of its actions for cardiovascular effects such as Praman et al. [63] evaluating* Tinospora crispa*, which suggested that the hypotensive effect was probably due to the action of the component against *β*2-adrenergic receptors. Dimo et al. [64] studying* Bidens pilosa* extracts justified the hypotensive response through the *β*-adrenergic vasodilation.

## 4. Conclusions

The chemical composition of* E. falcata* and* E. crista-galli* species is based on alkaloids. Through the UPLC-MS analysis the presence of five different alkaloids can be suggested for the studied species, namely, erythristemine, 11*β*-methoxyglucoerysodine, erysothiopine, 11*β*-hydroxyerysodine-glucose, and 11-hydroxyerysotinone-rhanmoside. The hydroethanolic and aqueous extracts of* E. falcata* show to possess* in vivo* dose-dependent hypotensive activity, which could be associated with the alkaloids content. Besides, the mechanism of this effect may be related to the route of *β*-adrenergic receptors.

## Supplementary Material

MS spectra obtained from analysis of *E. falcata* and *E. crista-galli* extracts, whose chemical composition has been suggested with focus on alkaloidal constituents.

## Figures and Tables

**Table 1:** Phenolic and flavonoids content in leaves extracts obtained from *E. falcata *and *E. crista-galli*.

Extraction method	Total phenolic^*∗*^ mg GAE/mL (RSD%)	Total flavonoids^*∗*^ mgRE/g (RSD%)
*E. falcata*		
Infusion	0.8771 (3.80)	9.3471 (1.27)
Maceration	1.3193 (4.80)	7.7829 (0.85)
*E. crista-galli*		
Infusion	0.9506 (5.09)	10.4765 (1.50)
Maceration	1.4989 (4.29)	8.1976 (1.29)

^*∗*^The extracts were analyzed in triplicate.

**Table 2:** Chemical constituents of *E. falcata *and *E. crista-galli* identified by UPLC-MS with correspondent retention times, quasi-molecular ions in positive mode, and key fragments.

Species	Rt (min)	Molecular formula	[M+H]^+^ (*m/z*)	MS fragmentation [M+H]^+^ (*m/z*)	Compound
*E. falcata*					
1	11.72	C_20_H_25_O_4_N	344	316, 299	Erythristemine
2	12.17	C_25_H_33_O_9_N	492	478, 316, 299	11*β*-Methoxyglucoerysodine
3	12.95	C_19_H_21_O_7_NS	408	348, 316	Erysothiopine
4	13.58	C_24_H_31_O_9_N	478	316, 299	11*β*-Hydroxyerysodine-glucose
5	13.94	C_24_H_31_O_9_N	478	316, 299	11-Hydroxyerysotinone-rhamnoside
*E. crista-galli*					
1	11.5	C_20_H_25_O_4_N	344	316, 299	Erythristemine
3	12.94	C_19_H_21_O_7_NS	408	349, 332, 316	Erysothiopine
4	13.42	C_24_H_31_O_9_N	478	316, 299	11*β*-Hydroxyerysodine-glucose
5	13.81	C_24_H_31_O_9_N	478	332, 316, 299	11-Hydroxyerysotinone-rhamnoside
