# Supplementary material for: Alkaloids in Erythrina by UPLC-ESI-MS and In Vivo Hypotensive Potential of Extractive Preparations
Source: Evid Based Complement Alternat Med. 2015 Aug 18;2015:959081. doi: 10.1155/2015/959081 (PMC4556073; doi:10.1155/2015/959081)
Supplement: Supplementary file 1 — MS spectra obtained from analysis of E. falcata and E. crista-galli extracts, whose chemical composition has been suggested with focus on alkaloidal constituents. [file 959081.f1.pdf]

SUPPLEMENTARY MATERIAL

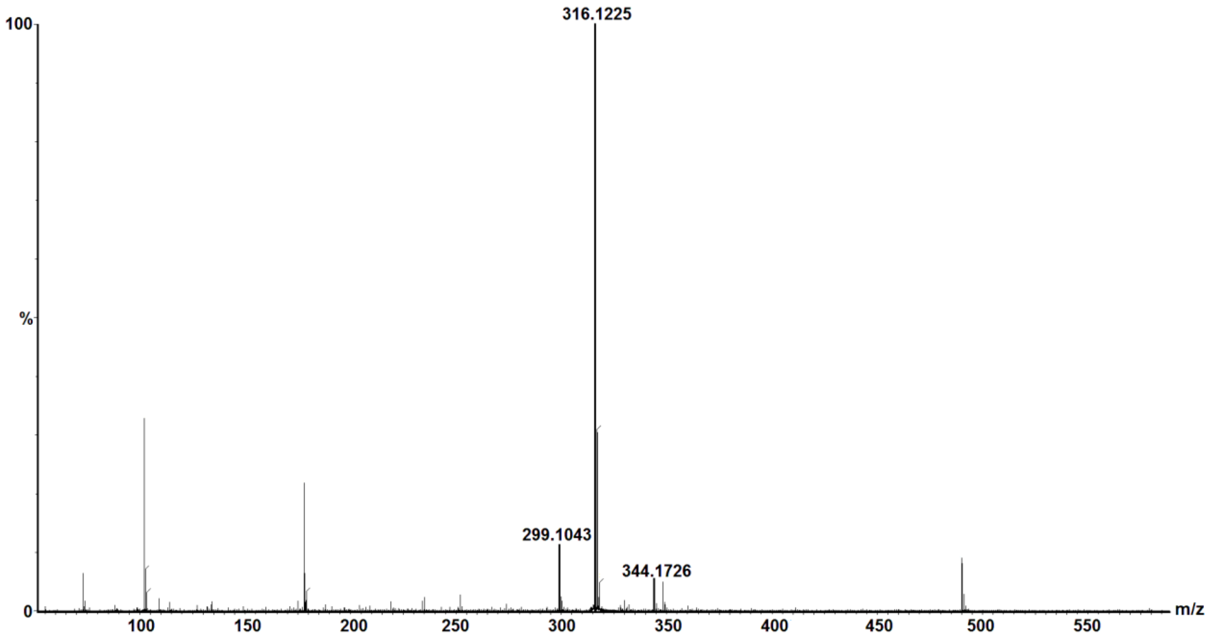

Figure 1. MS spectrum of Erythristemine in *E. falcata*.

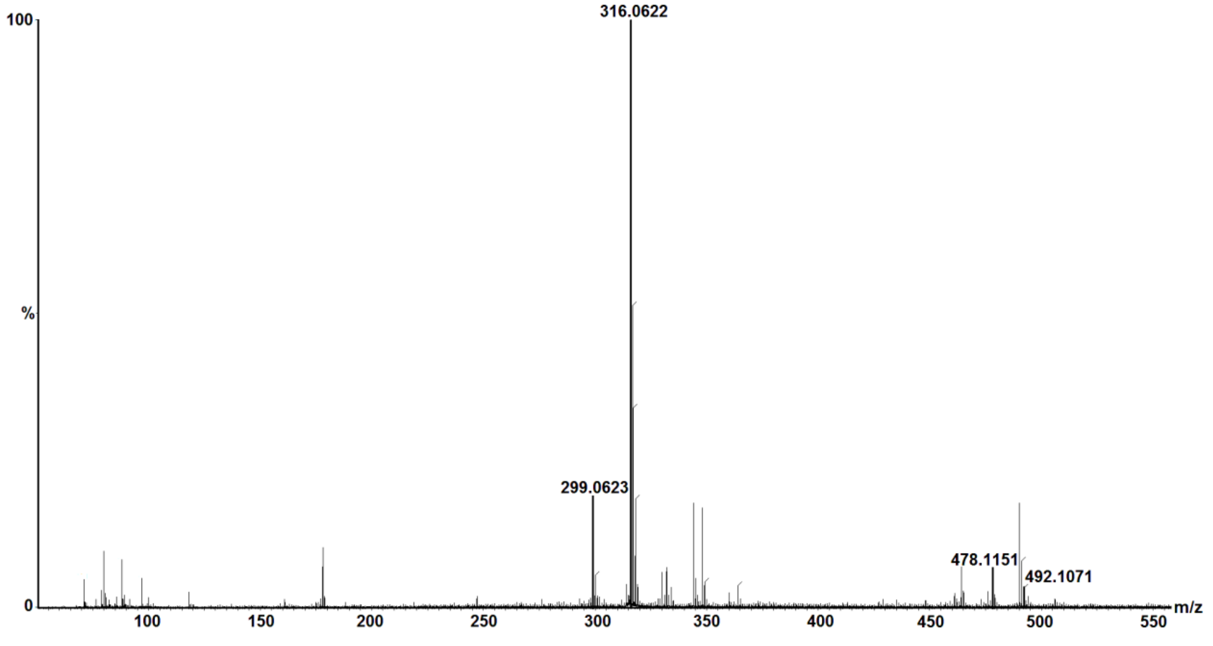

Figure 2. MS spectrum of 11β-Methoxyglucoerysodine in *E. falcata*.

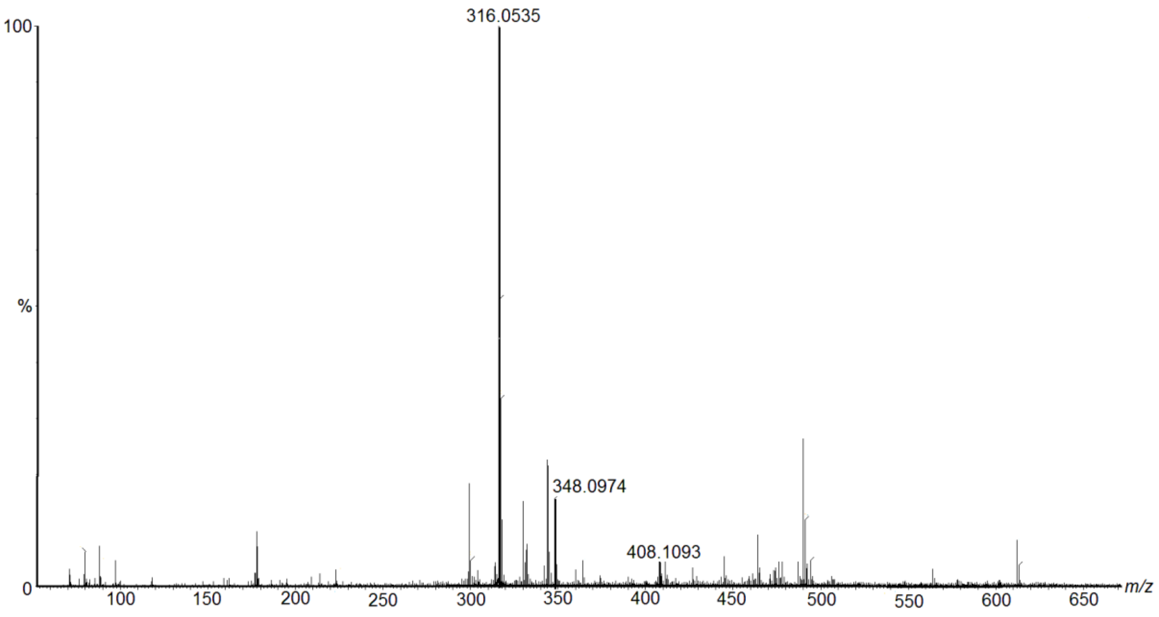

Figure 3. MS spectrum of Erysothiopine in *E. falcata*.

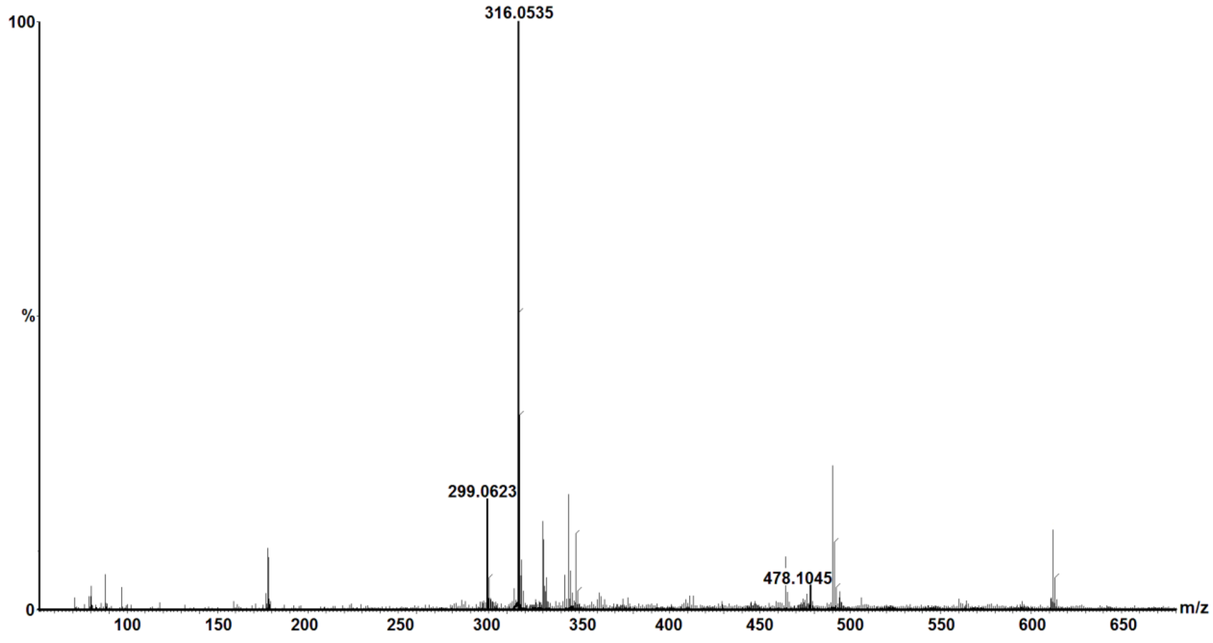

Figure 4. MS spectrum of 11β-Hydroxyerysodine-glucose in *E. falcata*.

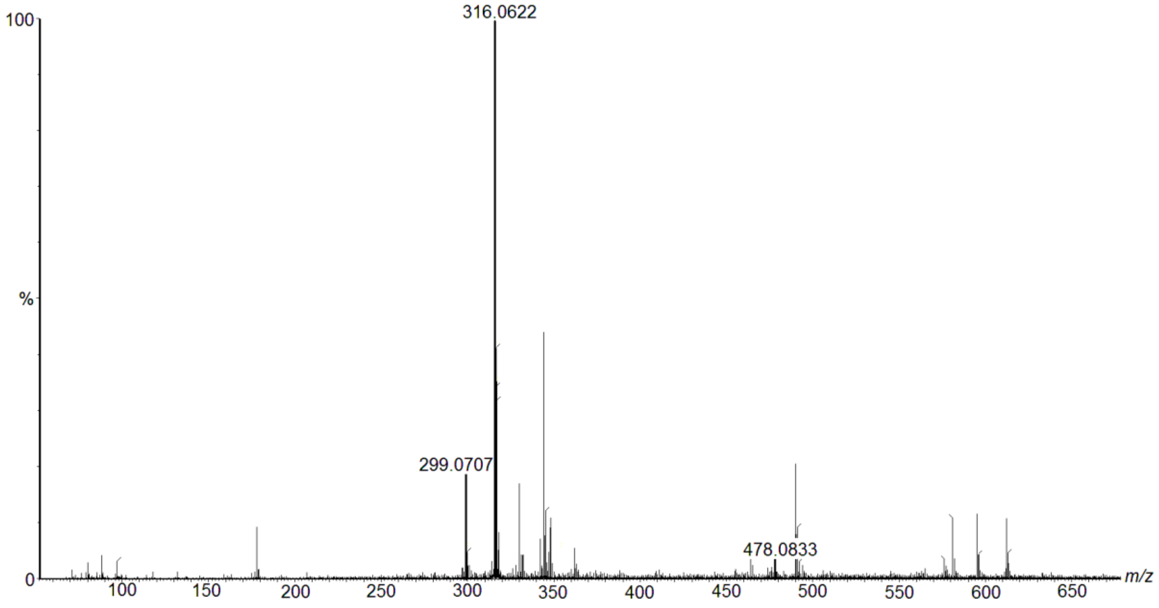

Figure 5. MS spectrum of 11-Hydroxyerysotinone-ranmoside in *E. falcata*.

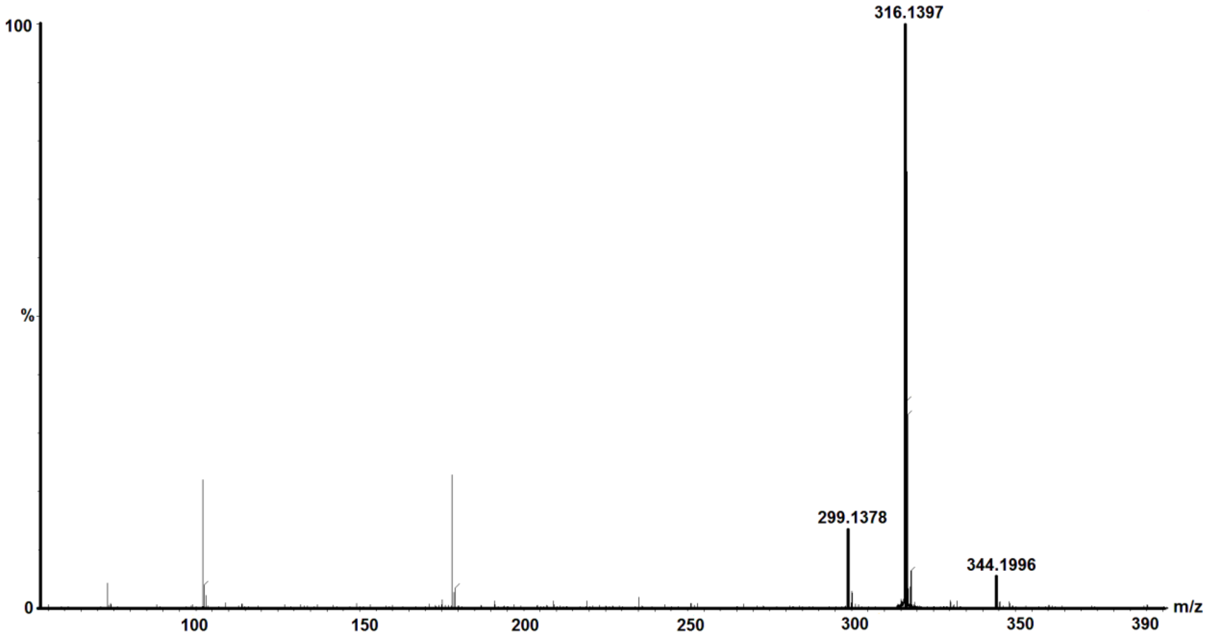

Figure 6. MS spectrum of Erythristemine in *E. crista-galli*.

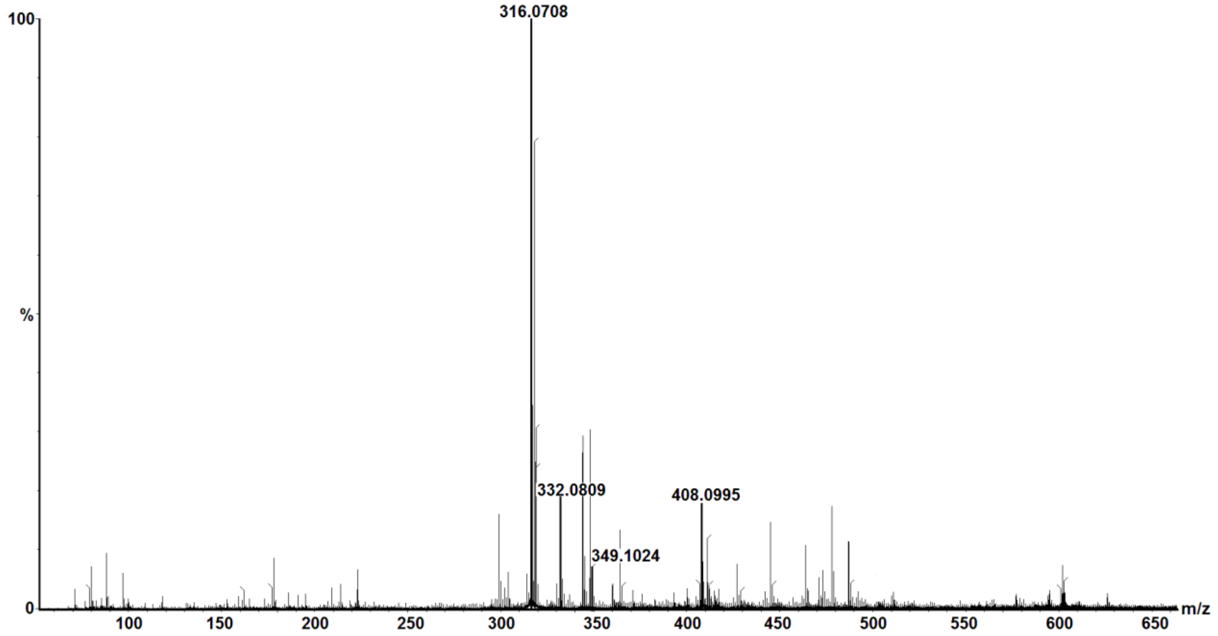

Figure 7. MS spectrum of Erysothiopine in *E. crista-galli*.

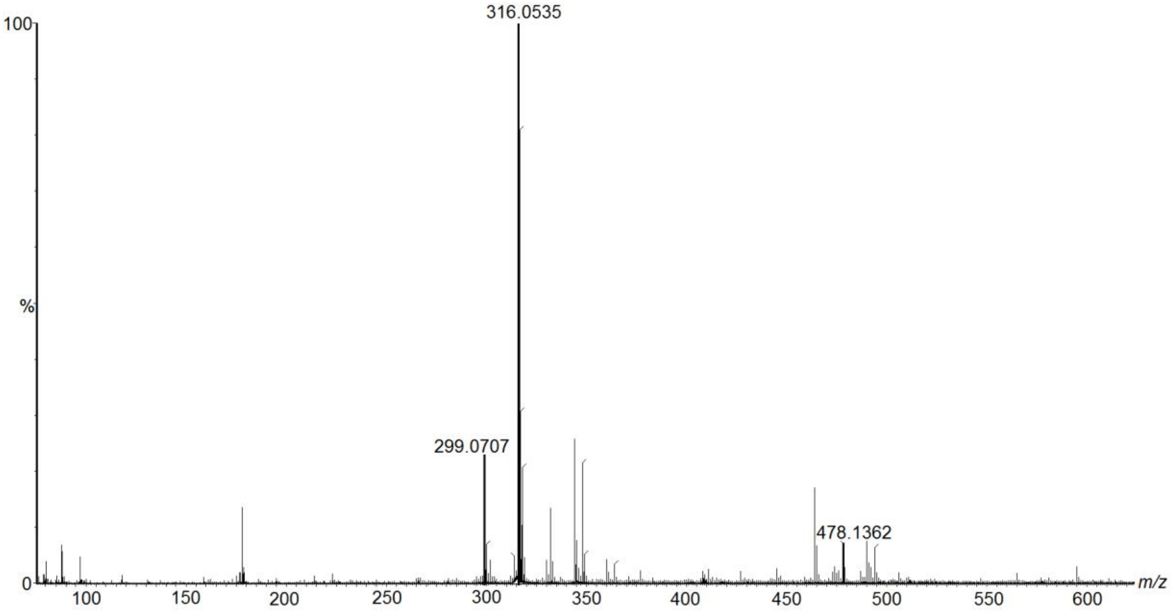

Figure 8. MS spectrum of 11β-Hydroxyerysodine-glucose in *E. crista-galli*.

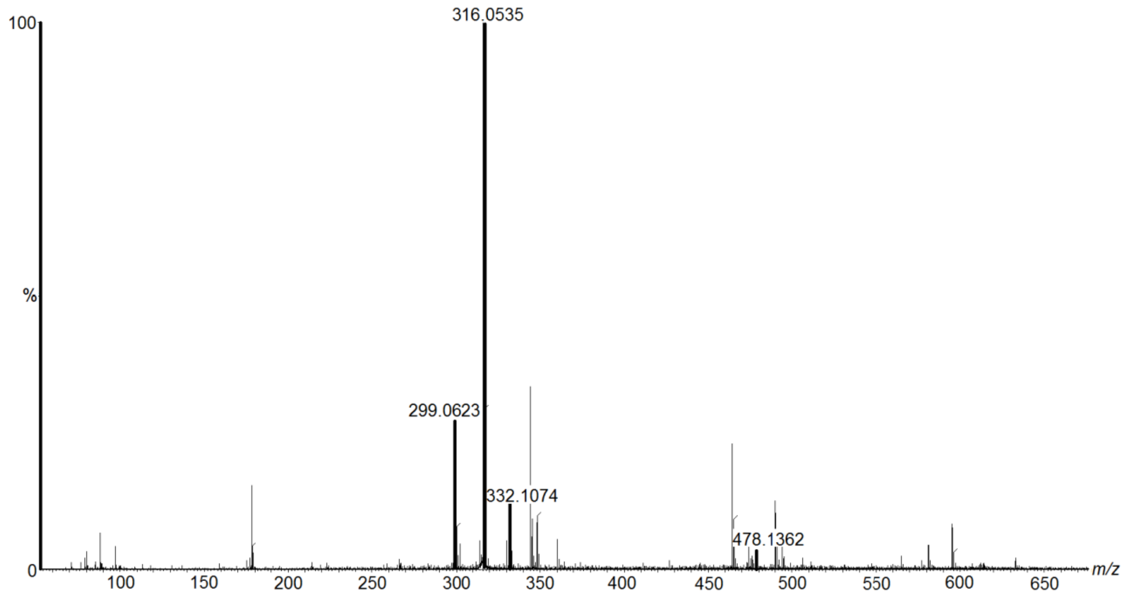

Figure 9. MS spectrum of 11-Hydroxyerysotinone-ranmoside in *E. crista-galli*.
